# Longitudinal integrated analysis of gut microbiota and metabolites in rectal cancer patients undergoing chemoradiotherapy

**DOI:** 10.3389/fmicb.2026.1934703

**Published:** 2026-10-14

**Authors:** Lin Wang, Pei Zhang, Yujing Zhang, Yan Li, Baolin Qu, Lihua Peng

**Affiliations:** 1 Microbiota Laboratory, Clinical Division of Microbiota, Department of Gastroenterology and Hepatology, The First Medical Center of Chinese PLA General Hospital, Beijing, China; 2 Chinese PLA Medical School, Beijing, China; 3 Department of Radiation Oncology, The First Medical Center of Chinese PLA General Hospital, Beijing, China

**Keywords:** colorectal cancer, gut microbiota, metabolites, pelvic chemoradiotherapy, temporal analysis

## Abstract

**Background:**

Longitudinal changes in the fecal microbiome and metabolome during concurrent chemoradiotherapy (CCRT) for rectal cancer remain incompletely characterized.

**Methods:**

In this prospective single-center cohort, 19 patients with rectal cancer were enrolled and 17 were included in the longitudinal analysis after two prespecified sampling-window exclusions. Fecal specimens were scheduled at nine time points: R0, collected before the first dose of capecitabine and before radiotherapy, and R3 through R24 at three-fraction intervals during treatment. Twenty unmatched healthy volunteers provided one fecal specimen for a descriptive pretreatment comparison. Full-length 16S rRNA gene sequencing and untargeted metabolomics were performed. Longitudinal microbial counts were analyzed using negative-binomial generalized linear mixed models, and metabolite features were analyzed using linear mixed-effects models; participant was included as a random intercept, and *P* values were adjusted using the Benjamini–Hochberg false discovery rate (FDR).

**Results:**

Nineteen genera and 27 of 176 analyzed species showed significant linear time trends during CCRT (FDR < 0.05). *Parvimonas micra, Peptostreptococcus stomatis*, and *Gemella morbillorum* decreased, whereas *Lachnoclostridium pacaense, Blautia hydrogenotrophica, [Eubacterium] sulci*, and *Coprococcus eutactus* increased. Of 2,873 analyzed metabolic features, 393 changed significantly over time. In a secondary same-cohort analysis, 17 of 50 correlation-selected taxon-metabolite pairs remained significant in time-adjusted mixed-effects models, including a positive association between *C. eutactus* and a feature annotated as stercobilin. Among 103 significant features with KEGG identifiers, three pathways met a nominal raw-P threshold, but none remained significant after FDR correction. Pretreatment patient samples had lower Shannon diversity and differed from healthy-control samples by ANOSIM; these comparisons were descriptive because the groups were not matched for age or sex. Five species shared the same case-control direction in this cohort and GMrepo reports, but only *P. stomatis* and *G. morbillorum* also showed significant longitudinal declines during CCRT.

**Conclusion:**

This small, intensively sampled cohort identifies exploratory fecal microbial and metabolic trajectories during capecitabine-based CCRT. The findings do not establish causal mechanisms, microbial functions, disease specificity, or biomarker performance and require independent confirmation in larger cohorts.

## 1 Introduction

Colorectal cancer ranks as the third most common cancer worldwide, with a global incidence of 10% and a mortality rate of 9.4%, and its incidence among younger patients is rising (Klimeck et al., 2023). The gut microbiome plays a crucial role in maintaining the mucosal barrier and homeostasis (Yin et al., 2023). By producing metabolites and toxins that influence cell proliferation, regulate inflammation and immunity, and induce DNA damage, gut microbes participate in gastrointestinal tumorigenesis, tumor progression, and treatment outcomes (Fu et al., 2021). Dysbiosis of gut microbiota is a key driver in the development and progression of colorectal cancer (Zhou et al., 2026). For example, *Clostridium difficile* can promote tumor progression by mediating immune suppression and enhancing cancer cell proliferation, while colicin-producing *Escherichia coli* can directly participate in carcinogenesis by inducing DNA damage (Anderson and Sears, 2023). These microbial factors are also significantly associated with the efficacy and drug resistance of radiotherapy and chemotherapy in rectal cancer (Taoum et al., 2025). Adjuvant chemoradiotherapy for locally advanced rectal cancer significantly reshapes the gut microbiome, and radiotherapy leads to a sustained reduction in microbial diversity during treatment by altering microbial composition, mucosal immunity, and the systemic immune system (Sun et al., 2023). However, the temporal dynamics of microbiota and metabolites throughout adjuvant chemoradiotherapy for rectal cancer remain poorly understood, and the evolutionary trajectory of colorectal cancer-associated microbiota under radiation stress and its functional metabolic responses remain unclear (Zhou et al., 2025). This study therefore designed a prospective longitudinal investigation enrolling rectal cancer patients receiving radiotherapy combined with capecitabine. The core objectives were to systematically analyze the temporal patterns of gut microbiota changes during chemoradiotherapy and the associated metabolic alterations, and to screen colorectal cancer-associated microbiota using public databases and longitudinally track their dynamic patterns and metabolic characteristics within this cohort.

## 2 Materials and methods

### 2.1 Study participants

The study included patients undergoing chemoradiotherapy for rectal cancer at the First Medical Center of the PLA General Hospital from August 2022 to August 2023, along with 20 healthy volunteers. Inclusion criteria are as follows: (1) Patients with pathologically confirmed rectal cancer diagnosed by colonoscopy, who are scheduled to undergo pelvic radiotherapy concurrent with oral capecitabine; (2) Aged 18–75 years, regardless of gender; (3) Voluntarily participating in this study after signing an informed consent form, and able to cooperate with sampling and follow-up. Exclusion criteria are as follows: (1) Undergone bowel cleansing procedures or taken antibiotics or other medications affecting gut microbiota within the past month; (2) Active infections within the past 3 months or currently present, including mycobacterial infections and opportunistic infections; (3) Received vaccinations within the past 3 months, during the study period, or within 6 months after the last study dose; (4) Pregnant or lactating women.

### 2.2 Study design

Based on the patient's condition and in accordance with the Chinese Guidelines for the Diagnosis and Treatment of Colorectal Cancer (D. Republic Of China Chinese Medical Association Oncology Branch, 2025), body positioning was performed using a frame, followed by CT localization to develop the radiotherapy plan. Volumetric modulated arc therapy (VMAT) combined with image-guided radiotherapy (IGRT) was administered. Radiation therapy was delivered to different target volumes based on the patient's condition, with a pelvic dose of 45–50.4 Gy in 25–28 fractions and a single fraction dose of 1.8–2.0 Gy. Two weeks prior to initiating radiotherapy and during the treatment period, the patient received oral chemotherapy with capecitabine tablets at 825 mg/m^2^ twice daily, five days per week. This study was approved by the Ethics Committee of the First Medical Center, PLA General Hospital (S2022-301-01).

### 2.3 Microbiome data processing

Microbial community genomic DNA was extracted from fecal samples using the E.Z.N.A.^®^ soil DNA Kit (Omega Bio-tek, Norcross, GA, USA) according to the manufacturer's instructions. DNA concentration and purity were determined using a NanoDrop2000 spectrophotometer (Thermo Scientific, USA). The bacterial 16S rRNA gene was amplified with the universal primers 27F (5′-AGRGTTYGATYMTGGCTCAG-3′) and 1492R (5′-RGYTACCTTGTTACGACTT-3′) (Callahan et al., 2019), tailed with PacBio barcode sequences. PCR reactions (20 μL) contained 5 × FastPfu buffer (4 μL), 2.5 mM dNTPs (2 μL), each primer (5 μM, 0.8 μL), FastPfu DNA Polymerase (0.4 μL), and template DNA (10 ng). Amplification conditions were as follows: 95 °C for 3 min; 27 cycles of 95 °C for 30 s, 60 °C for 30 s, and 72 °C for 45 s; and 72 °C for 10 min. PCR products were purified using AMPure^®^ PB beads (Pacific Biosciences, CA, USA) and quantified with a Qubit 4.0 fluorometer (Thermo Fisher Scientific, USA). SMRTbell libraries were constructed using the SMRTbell Prep Kit 3.0 (Pacific Biosciences, CA, USA) and sequenced on the PacBio Sequel IIe System (Pacific Biosciences, CA, USA) by Majorbio Bio-Pharm Technology Co. Ltd (Callahan et al., 2019; Wenger et al., 2019). (Shanghai, China). HiFi reads were generated via SMRT Link v11.0 (Wenger et al., 2019).

Full-length 16S rRNA gene sequencing data were processed using the Majorbio Cloud Platform (https://cloud.majorbio.com). HiFi reads were barcode-identified and length-filtered, and 16S rRNA gene sequences shorter than 1,000 bp or longer than 1,800 bp were removed. Raw reads were quality-filtered using fastp (v0.19.6) (Chen et al., 2018). Reads were denoised using DADA2(Callahan et al., 2016) in QIIME2 (version 2020.2) (Bolyen et al., 2019) with parameters optimized for PacBio full-length 16S reads to generate amplicon sequence variants (ASVs). Sequences were rarefied to 6,000 reads per sample, yielding an average Good's coverage of 99.68%. Taxonomic assignment of ASVs was performed using the Naive Bayes classifier (Bokulich et al., 2018) in QIIME2 against the SILVA (Quast et al., 2012) 16S rRNA database (v138). Species-level relative abundance profiles were used for downstream analyses. Fecal samples were collected from patients longitudinally and grouped by treatment stage: pre-radiotherapy baseline (R0) and subsequent intervals after every three radiotherapy fractions (R3, R6, R9, R12, R15, R18, R21, and R24). For the 20 healthy volunteers, a single fecal sample was collected from each individual at enrollment. All samples were processed using the same protocol. Species present in < 10% of all samples were excluded, leaving 176 species for statistical modeling.

### 2.4 Metabolomic data processing

#### 2.4.1 Sample preparation

Fecal samples (50 ± 5 mg) were extracted with 400 μL of methanol:water (4:1, v/v) containing four internal standards (including L-2-chlorophenylalanine at 0.02 mg/mL). Samples were ground in a cryogenic grinder (−10 °C, 50 Hz, 6 min), sonicated (5 °C, 40 kHz, 30 min), incubated at −20 °C for 30 min, and centrifuged (13,000 g, 4 °C, 15 min). The supernatant was collected for LC-MS analysis.

#### 2.4.2 LC-MS analysis

LC-MS analysis was performed on a Thermo Scientific UHPLC-Q Exactive HF-X system equipped with an ACQUITY UPLC HSS T3 column [100 mm × 2.1 mm i.d., 1.8 μm; Waters (Waters Corporation, Milford, MA, USA)]. Mobile phase A was 95% water with 5% acetonitrile (containing 0.1% formic acid), and mobile phase B was 47.5% acetonitrile, 47.5% isopropanol, and 5% water (containing 0.1% formic acid). The injection volume was 3 μL, and the column temperature was 40 °C. Mass spectra were acquired in both positive and negative ion modes with a scan range of *m*/*z* 70–1,050. The resolution was 60,000 for full MS and 7,500 for MS^2^, with normalized collision energies of 20, 40, and 60 eV.

#### 2.4.3 Quality control

Quality control (QC) samples were prepared by pooling equal volumes (20 μL) of supernatant from all samples and were injected every 5–15 samples throughout the run. Internal standard stability was assessed using z-score plots, and all internal standards remained within ±2 standard deviations, indicating acceptable analytical stability (Broadhurst et al., 2018).

#### 2.4.4 Data preprocessing and metabolite identification

Raw data were processed using Progenesis QI v3.0 (Waters) for peak extraction, alignment, and identification. Metabolites were annotated against the HMDB (Wishart et al., 2022), METLIN (Guijas et al., 2018), and an in-house database with a mass error threshold of < 10 ppm. Features with a missing rate >20% within each group were removed. For features with partial missing values, missing values were imputed using the minimum value across all samples. Data were normalized by total sum and standardized. Features with a relative standard deviation (RSD) >30% in QC samples were filtered out. Peak intensities were then log-transformed (base 10) and auto-scaled. After preprocessing, 11,882 metabolites were retained. Metabolites with a standard deviation < 1.0 across samples were further excluded, leaving 2,873 metabolites for statistical modeling. All samples were processed and analyzed in a single batch to avoid batch effects; no batch correction was applied.

#### 2.4.5 Metabolite identification confidence

Metabolites were reported as putatively annotated features unless Level 1 identification (authentic standard confirmation) was documented. Identification confidence levels were not systematically assigned in this study, and targeted validation of key metabolites (e.g., taurocholate, S-sulfotyrosine, chenodeoxycholic acid, stercobilin) was not performed. These compounds should be considered as putative annotations, and their identities require confirmation using authentic standards in future targeted analyses (Sumner et al., 2007).

### 2.5 Statistical analysis

All analyses were performed in R version 4.6.1(R Foundation for Statistical Computing, Vienna, Austria) (2026-06-24). As an exploratory approach, STEM (short time-series expression miner) (Ernst and Bar-Joseph, 2006) clustering was first applied to visualize species with similar temporal patterns. STEM results were used only to guide downstream analyses and should not be interpreted as statistical inference.

To formally identify species and genera with significant temporal trends, negative binomial generalized linear mixed models (GLMMs) were fitted using the glmmTMB package (version 1.1.14; CRAN, Vienna, Austria). Linear mixed-effects models (LMMs) were fitted using the lme4 package (version 2.0.6; CRAN, Vienna, Austria), with *P* values estimated using the lmerTest package (version 3.2.1; CRAN, Vienna, Austria).

For all models, time was modeled as an equally spaced numeric variable (0–8, representing each 3-fraction interval), assuming a linear temporal trend. Patient ID was included as a random intercept to account for within-subject correlations. For microbial count data, log (total reads per sample) was included as an offset to normalize for sequencing depth, and a negative binomial distribution using the nbinom2 parameterization with a log link was used to account for overdispersion:

ReadCount ~ Time + offset(log_total) + (1 | PatientID)

For metabolite data (log-transformed intensities), the model was:

Metabolite_LogValue ~ Time + (1 | PatientID)

For species–metabolite association analysis, the model was:

Metabolite_LogValue ~ Species + Time + (1 | PatientID)

To complement the species-level analysis, genus-level temporal trends were analyzed using the same negative binomial GLMM framework as described above. Species and genera with a prevalence < 10% across all samples were excluded prior to modeling. Metabolites with a standard deviation < 1.0 across samples were excluded to remove uninformative features. *P* values were adjusted for multiple testing using the Benjamini–Hochberg false discovery rate (FDR) method within each analysis separately (i.e., all 176 species, all analyzed genera, all 2,873 metabolites, or all tested species–metabolite pairs), with FDR < 0.05 considered statistically significant.

KEGG pathway enrichment analysis was performed using MetaboAnalyst 6.0 (Pang et al., 2024) with the human KEGG pathway library (Ogata et al., 1999). The hypergeometric test was used for enrichment analysis, and relative-betweenness centrality was used for topology analysis. Pathways with raw *P* < 0.05 were considered significantly enriched (Xia and Wishart, 2010).

### 2.6 Microbiome comparison

Alpha diversity was assessed using the Shannon index at both the genus and species levels. The Shannon index was calculated for each sample using the vegan package (version 2.7.6) in R (version 4.6.1; R Foundation for Statistical Computing, Vienna, Austria). Differences in Shannon index between CRC patients and healthy controls were tested using the Wilcoxon rank-sum test, with *P* < 0.05 considered statistically significant.

Beta diversity was assessed using Bray-Curtis distance based on genus- and species-level abundance profiles using the vegan package (version 2.7.6; CRAN, Vienna, Austria). Principal coordinate analysis (PCoA) was performed to visualize the overall microbial community structure. Analysis of similarities (ANOSIM) with 999 permutations was used to test for significant differences in community composition between groups.

Differential abundance analysis was performed using Wilcoxon rank-sum tests with Benjamini–Hochberg FDR correction using the rstatix package (version 1.1.0; CRAN, Vienna, Austria), with FDR < 0.05 considered statistically significant. In addition, LEfSe analysis (Segata et al., 2011) was performed using the Majorbio Cloud Platform (https://cloud.majorbio.com) to identify differentially abundant taxa between CRC patients and healthy controls. The analysis was performed at both the genus and species levels, with an LDA score threshold of 2.0 and a Kruskal–Wallis test *P*-value of 0.05.

### 2.7 Validation using GMrepo

The GMrepo database (version 2, accessed on 2025-11-19, https://gmrepo.humangut.info/) (Wu et al., 2020) is a curated repository of human gut microbiome data that integrates published studies and provides information on taxon-disease associations. We used it to validate whether the differentially abundant taxa identified in our cohort were consistent with previously reported CRC-associated taxa. To validate our findings against external databases, differential genera and species identified in our cohort using Wilcoxon rank-sum tests with Benjamini–Hochberg FDR correction [rstatix package, version 1.1.0 (CRAN, Vienna, Austria), FDR < 0.05] were compared with CRC-associated taxa curated from published studies in the GMrepo database (https://gmrepo.humangut.info/). Taxa were classified into four categories based on the direction consistency between our cohort and GMrepo: “consistent” (same direction in both), “opposite” (opposite direction), “partial” (controversial in GMrepo), and “not validated” (not present in GMrepo). For species-level comparison, species names were standardized by replacing spaces with underscores to ensure accurate matching.

### 2.8 Exploratory longitudinal analysis

To explore the temporal dynamics of the 5 CRC-associated species validated against GMrepo (*Adlercreutzia equolifaciens, Peptostreptococcus stomatis, Gemella morbillorum, Haemophilus parainfluenzae*, and *Phocaeicola plebeius*) during CCRT, negative binomial GLMMs were fitted using the same framework as described in the “Statistical analysis” section. *P*-values were adjusted using the Benjamini–Hochberg FDR method, with FDR < 0.05 considered statistically significant. Given the small sample size (*n* = 17) and limited statistical power, this analysis should be interpreted as exploratory and hypothesis-generating.

## 3 Results

### 3.1 Patient and sample information

Nineteen patients with histologically confirmed rectal adenocarcinoma were enrolled. Two patients were excluded from the longitudinal analysis because a fecal specimen could not be obtained within the prespecified window spanning three radiotherapy fractions. The final longitudinal cohort therefore comprised 17 patients. The R0 specimen was collected before the first dose of capecitabine and before the initiation of radiotherapy. The 17 analyzed patients had a mean age of 57 ± 10 years; 12 (70.6%) were male and five (29.4%) were female. The mean radiation dose was 50.08 Gy. The descriptive comparison group included 20 healthy volunteers with a mean age of 35 ± 5 years; 17 (85.0%) were male and three (15.0%) were female. The marked age difference and the unmatched design were considered when interpreting patient-control comparisons.

### 3.2 Longitudinal multi-omics dynamics during CCRT

#### 3.2.1 Genus-level temporal dynamics

Negative-binomial generalized linear mixed models identified significant linear time trends for 19 genera after FDR correction: 8 increased and 11 decreased during CCRT (Supplementary Figure S1). Among the increasing genera, the model coefficients were 0.390 for *Lachnoclostridium* (FDR = 7.53 × 10^−4^), 0.087 for *Mediterraneibacter* (FDR = 1.54 × 10^**−3**^), and 0.387 for *Megamonas* (FDR = 6.62 × 10^−3^). Among the decreasing genera, the coefficients were −0.589 for *Peptostreptococcus* (FDR = 5.13 × 10^−6^), −0.406 for *Gemella* (FDR = 1.25 × 10^−5^), and −0.737 for *Parvimonas* (FDR = 1.63 × 10^−5^). Representative trajectories are shown in Supplementary Figure S2. These results describe changes in sequencing-depth-adjusted counts over the modeled treatment intervals and do not establish changes in absolute bacterial load.

#### 3.2.2 Species-level temporal dynamics and cross-validation

Short Time-series Expression Miner (STEM) clustering was used only to visualize groups of species with similar temporal patterns (Supplementary Figure S3). Formal inference was based on negative-binomial generalized linear mixed models. Of the 176 species that passed the prevalence filter, 27 showed significant linear time trends after FDR correction; 16 increased and 11 decreased (Figure 1).

**Figure 1:**
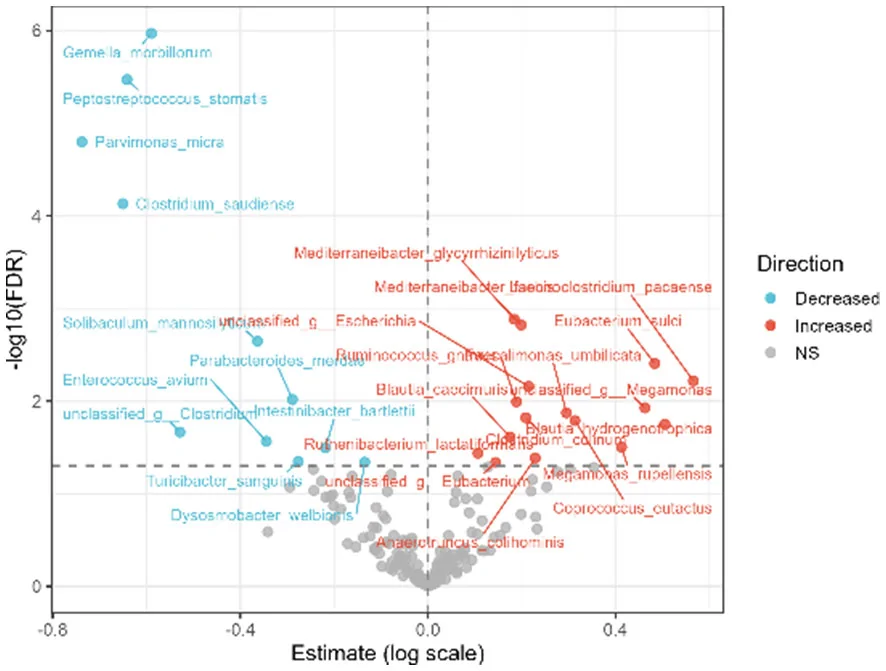
Volcano plot of microbial species with significant temporal trends during concurrent chemoradiotherapy (CCRT). Volcano plot showing the temporal trends of 176 microbial species during CCRT, analyzed using negative binomial generalized linear mixed models (GLMMs) with patient ID as a random intercept and log(total reads per sample) as an offset to normalize for sequencing depth. *P* values were adjusted using the Benjamini–Hochberg false discovery rate (FDR) method. The x-axis represents the effect size (estimate on the log scale, representing the change per 3-fraction interval), and the y-axis represents –log10(FDR). Each point represents one species. Red points indicate species with positive time coefficients and FDR < 0.05, blue points indicate species with negative time coefficients and FDR < 0.05, and gray points indicate non-significant species. Dashed lines indicate the significance threshold (FDR = 0.05). The 20 species with the smallest FDR values are labeled. A total of 27 species showed significant linear time trends (16 with positive coefficients and 11 with negative coefficients).

Species with negative time coefficients included *Parvimonas micra* (estimate = −0.738, FDR = 1.59 × 10^−5^), *Peptostreptococcus stomatis* (estimate = −0.641, FDR = 3.38 × 10^−6^), and *Gemella morbillorum* (estimate = −0.589, FDR = 1.07 × 10^−6^). Species with positive time coefficients included *Lachnoclostridium pacaense* (estimate = 0.566, FDR = 6.06 × 10^**−3**^), *Blautia hydrogenotrophica* (estimate = 0.505, FDR = 1.78 × 10^**−2**^), *[Eubacterium] sulci* (estimate = 0.483, FDR = 3.93 × 10^**−3**^), and *Coprococcus eutactus* (estimate = 0.313, FDR = 1.63 × 10^**−2**^; Figure 2). These labels identify taxonomic trajectories; functional properties were not measured by 16S rRNA sequencing.

**Figure 2:**
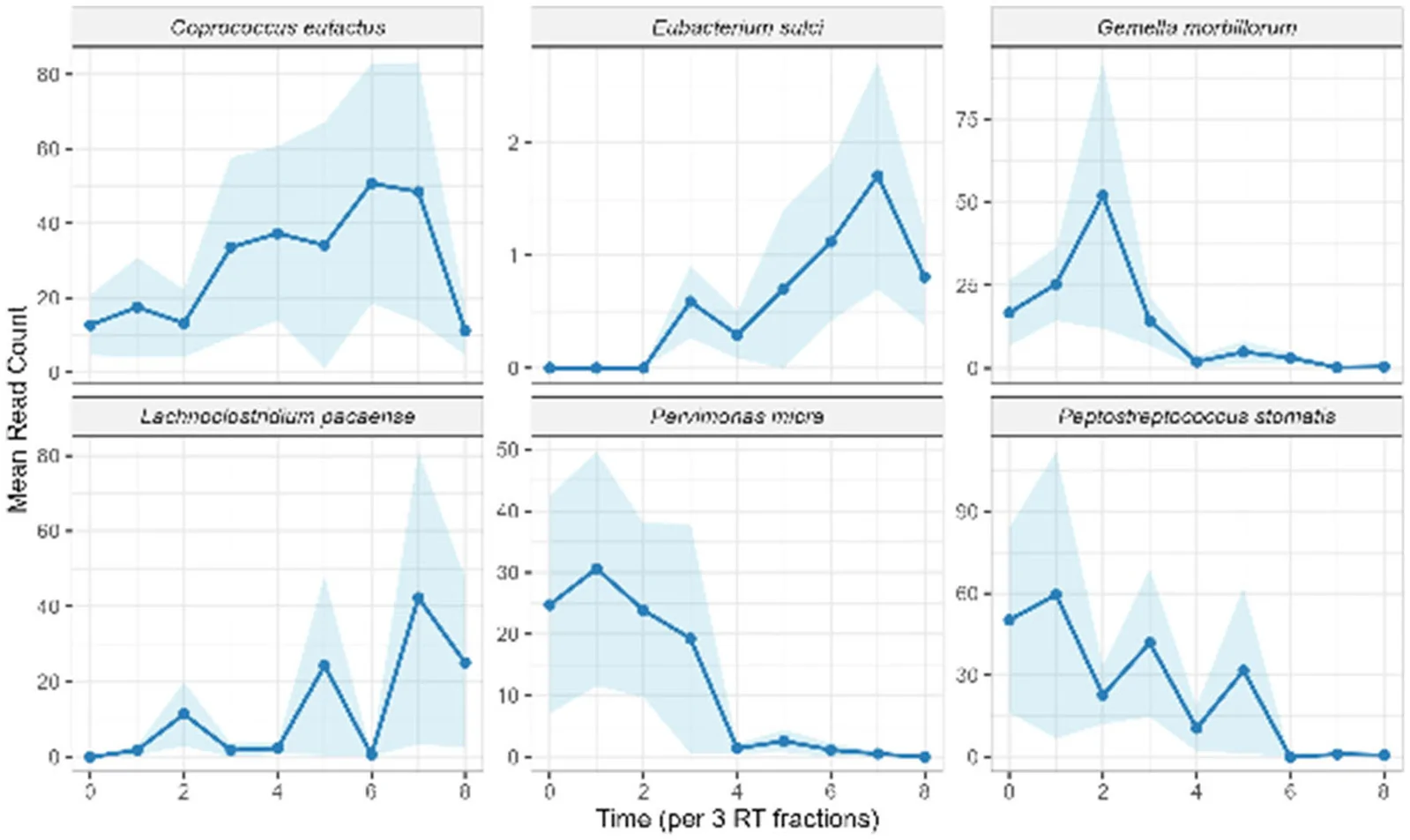
The six species shown are representative of the 27 species with significant temporal trends (FDR < 0.05). *Coprococcus eutactus*, [Eubacterium] sulci, and *Lachnoclostridium pacaense* exhibited significant increases over the treatment course, whereas *Parvimonas micra, Peptostreptococcus stomatis*, and *Gemella morbillorum* exhibited significant declines.

Fifteen genera had significant and directionally concordant genus- and species-level trends (Figure 1, Supplementary Figure S1, Supplementary Table S1). *Parvimonas, Peptostreptococcus, Gemella, Clostridium, Solibaculum, Parabacteroides, Intestinibacter, Turicibacter*, and *Dysosmobacter* decreased, whereas *Lachnoclostridium, Mediterraneibacter, Megamonas, Escherichia, Faecalimonas*, and *Ruthenibacterium* increased. No discordant direction was observed among these 15 genera.

Four additional genera showed significant genus-level trends without a significant species-level trend: *Peptoniphilus* (estimate = −0.557, FDR = 3.64 × 10^**−3**^) and *Alistipes* (estimate = −0.122, FDR = 4.69 × 10^**−2**^) decreased, whereas *Tyzzerella* (estimate = 0.186, FDR = 1.62 × 10^**−2**^) and *unclassified Lachnospiraceae* (estimate = 0.077, FDR = 1.03 × 10^**−2**^) increased.

#### 3.2.3 Temporal dynamics of gut metabolites during CCRT

Of the 2,873 metabolic features included in the mixed-effects analysis, 393 showed significant linear time trends after FDR correction: 147 increased and 246 decreased (Figure 3). Features with prominent negative coefficients included those annotated as glucosyringic acid (estimate = −0.130, FDR = 1.73 × 10^−6^), 4-hydroxyestrone-2-S-glutathione (estimate = −0.150, FDR = 2.90 × 10^−6^), and cyclopassifloic acid A (estimate = −0.190, FDR = 7.70 × 10^−5^). Several features annotated as phospholipids, including PS (18:2/14:0), PC (LTE4/15:0), and DG (18:4/14:1), also had negative time coefficients.

**Figure 3:**
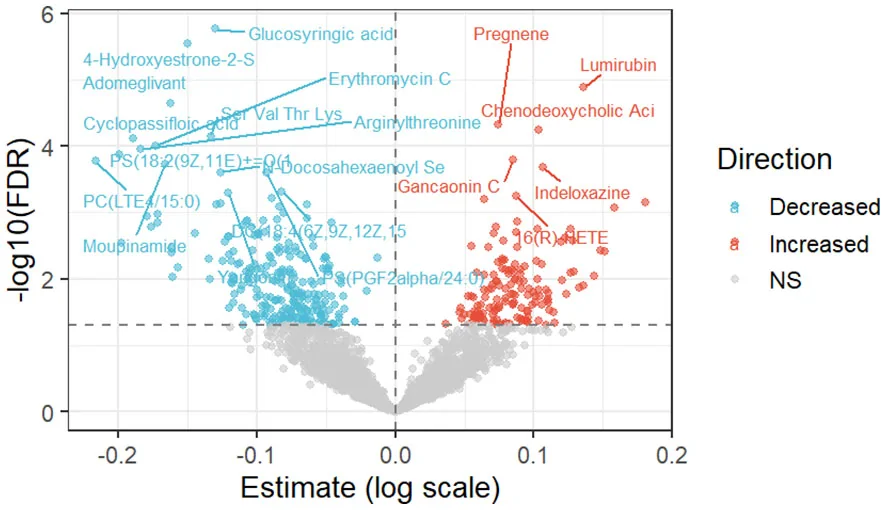
Volcano plot of metabolic features with significant temporal trends during CCRT. Volcano plot showing the temporal trends of 2,873 metabolic features during CCRT, analyzed using linear mixed-effects models (LMMs) with patient ID as a random intercept. *P* values were adjusted using the Benjamini–Hochberg false discovery rate (FDR) method. The x-axis represents the effect size (estimate on the log scale), and the y-axis represents –log10(FDR). Each point represents one metabolic feature. Red points indicate features with positive coefficients and FDR < 0.05, blue points indicate features with negative coefficients and FDR < 0.05, and gray points indicate non-significant features. Dashed lines indicate the significance threshold (FDR = 0.05). The 20 features with the smallest FDR values are labeled. A total of 393 metabolic features showed significant linear time trends (147 with positive coefficients and 246 with negative coefficients). Metabolite names are reported as putatively annotated features.

Features with prominent positive coefficients included those annotated as lumirubin (estimate = 0.136, FDR = 1.33 × 10^−5^), chenodeoxycholic acid (estimate = 0.103, FDR = 5.84 × 10^−5^), and pregnene (estimate = 0.074, FDR = 4.82 × 10^−5^). Because identification confidence and authentic-standard confirmation must be reported separately, these findings are described as annotated features unless level-1 identification is documented.

#### 3.2.4 Microbe-metabolite associations during CCRT

In the initial screening step, Spearman analysis identified 2,527 taxon-metabolite correlations with FDR < 0.05 (Supplementary Table S2). The 50 pairs with the largest absolute correlation coefficients (|r| > 0.5) were then examined in linear mixed-effects models that included time and a participant-specific random intercept. Seventeen of these 50 selected pairs remained significant after FDR correction (Figure 4). Because selection and mixed-model testing were conducted in the same cohort, this two-stage analysis is exploratory and does not constitute internal or external validation.

**Figure 4:**
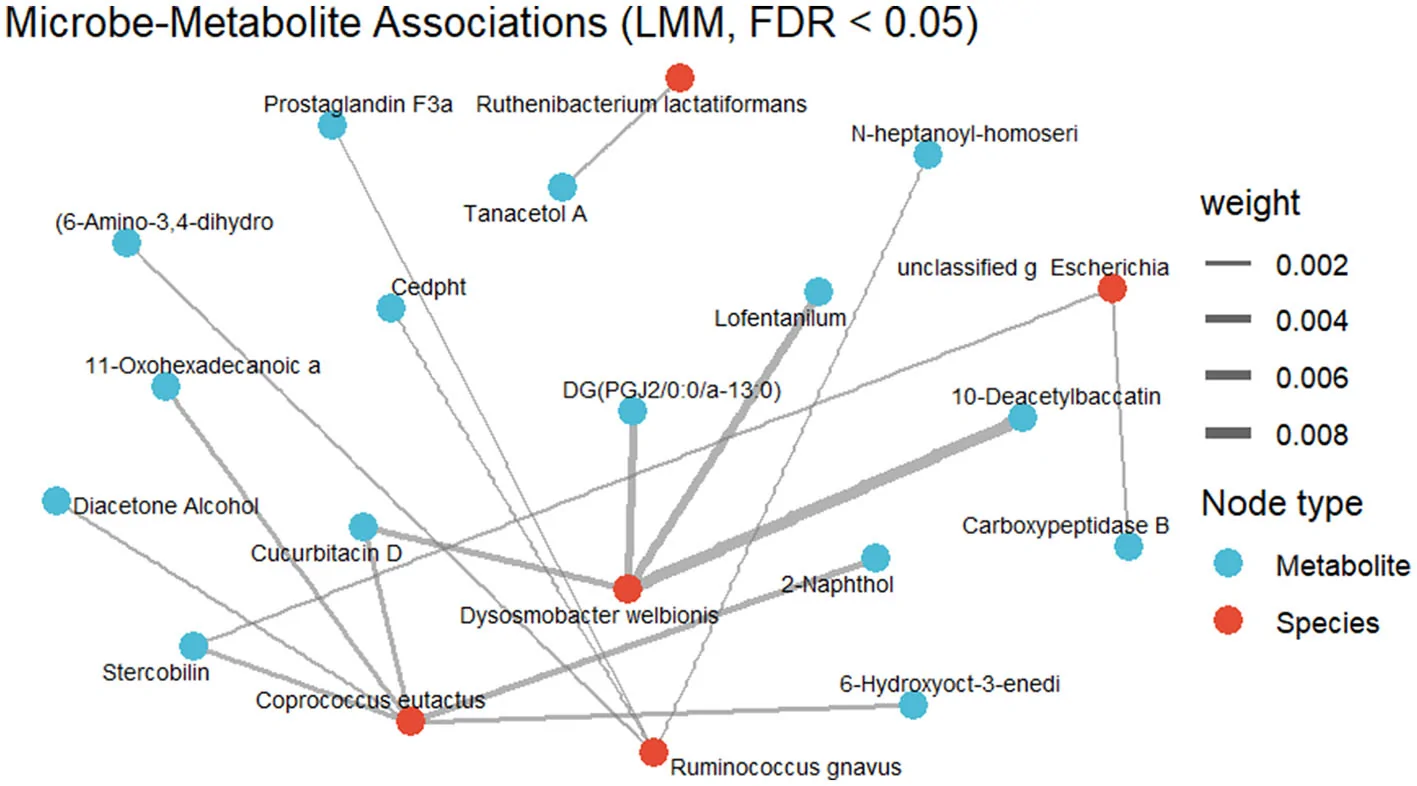
Microbe–metabolite association network during CCRT. Nodes represent microbial species (red) and metabolic features (blue). Edges represent significant associations identified by linear mixed-effects models (FDR < 0.05), with edge width proportional to the absolute effect size. The network includes 17 significant taxon–feature associations involving 5 microbial species and multiple metabolic features. Key associations include *Coprococcus eutactus* with a feature annotated as stercobilin, *Ruminococcus gnavus* with a feature annotated as prostaglandin F3a, and Escherichia with the stercobilin-annotated feature (opposite direction to *Coprococcus eutactus*). Because the candidate pairs were selected and tested in the same cohort, this analysis is exploratory and does not establish that any taxon produced or regulated the corresponding metabolite.

*C. eutactus* was positively associated with six metabolic features, including a feature annotated as stercobilin (estimate = 1.35 × 10^**−3**^, FDR = 3.81 × 10^**−2**^). *Ruminococcus gnavus* was negatively associated with a feature annotated as prostaglandin F3α (estimate = −4.93 × 10^−4^, FDR = 1.57 × 10^**−2**^). *Escherichia* was negatively associated with the stercobilin-annotated feature (estimate = −2.82 × 10^−5^, FDR = 4.81 × 10^**−2**^), opposite to the association observed for *C. eutactus*. These models identify statistical associations after adjustment for a linear time term; they do not show that the taxa produced or regulated the corresponding metabolites.

#### 3.2.5 KEGG pathway enrichment of treatment-responsive metabolites

Of the 393 metabolic features with significant time trends, 103 had KEGG identifiers and were included in pathway over-representation analysis. Three pathways met the nominal raw-*P* threshold: primary bile acid biosynthesis (4 hits, raw *P* = 0.0011, FDR = 0.088, impact = 0.024), glycerophospholipid metabolism (3 hits, raw *P* = 0.0058, FDR = 0.232, impact = 0.162), and glycine, serine, and threonine metabolism (2 hits, raw *P* = 0.0467, FDR = 1.000, impact = 0.048; Figure 5). None remained significant at FDR < 0.05. The pathway results are therefore reported as nominal exploratory signals. Figure 6 summarizes the observed taxon-metabolite associations and the nominal pathway annotations; it does not represent a demonstrated mechanistic axis.

**Figure 5:**
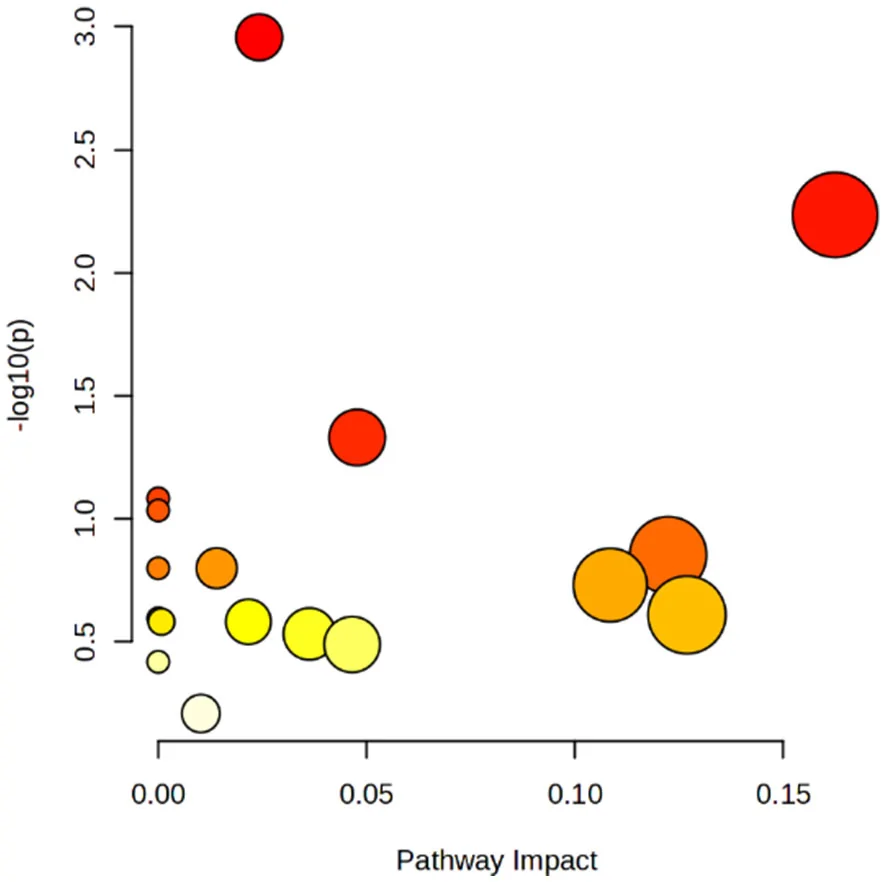
KEGG pathway over-representation analysis of metabolic features with significant time trends. Scatter plot showing the enriched pathways based on 103 of the 393 metabolic features with KEGG identifiers. The x-axis represents pathway impact (topological importance based on relative-betweenness centrality), and the y-axis represents –log10(raw *P*-value) from the hypergeometric test. Dot size is proportional to the number of matched features (Hits), and dot color indicates *P*-value (red = more significant, yellow = less significant). Three pathways were nominally enriched (raw *P* < 0.05): primary bile acid biosynthesis (*P* = 0.0011), glycerophospholipid metabolism (*P* = 0.0058), and glycine, serine and threonine metabolism (*P* = 0.0467). None remained significant after FDR correction. Pathway enrichment was performed using MetaboAnalyst 6.0 with the human KEGG pathway library.

**Figure 6:**
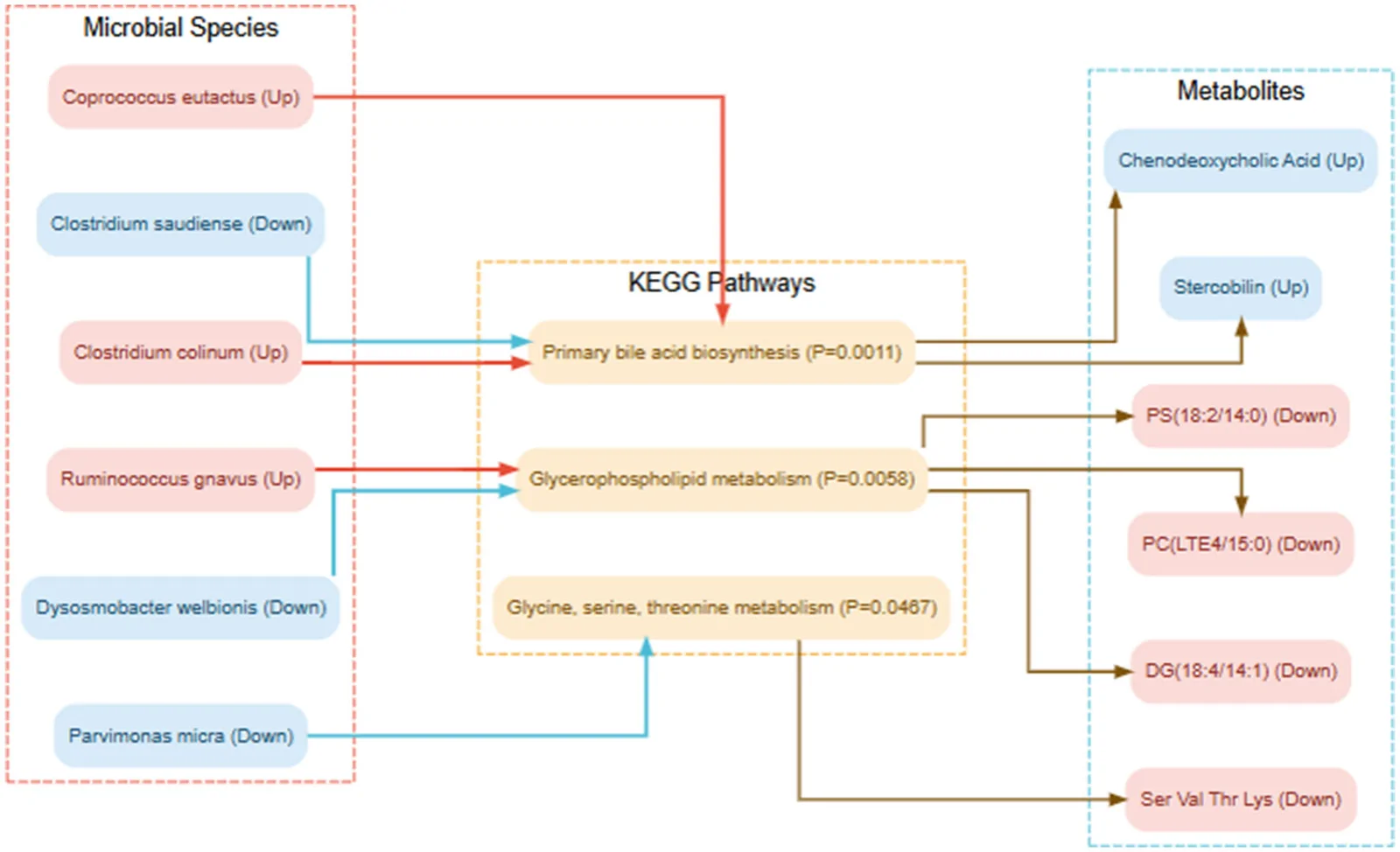
Exploratory microbe–pathway–metabolite association map during CCRT. The three-column diagram illustrates exploratory associations between microbial species, KEGG pathways, and metabolic features. Left column: microbial species with significant temporal trends (red = positive time coefficients, blue = negative time coefficients), including *Coprococcus eutactus, Clostridium saudiense, Clostridium colinum, Ruminococcus gnavus, Dysosmobacter welbionis*, and *Parvimonas micra*. Middle column: KEGG pathways nominally over-represented (raw *P* < 0.05), including primary bile acid biosynthesis (*P* = 0.0011), glycerophospholipid metabolism (*P* = 0.0058), and glycine, serine and threonine metabolism (*P* = 0.0467). Right column: metabolic features (red = negative time coefficients, blue = positive time coefficients), including features annotated as chenodeoxycholic acid, stercobilin, and multiple phospholipids (PS, PC, DG). Arrows indicate literature-reported functional associations between species and pathways, and KEGG-annotated links between pathways and features. This diagram is exploratory and does not represent a demonstrated mechanistic axis. None of the pathways remained significant after FDR correction.

### 3.3 Observing the temporal changes in gut microbiota and metabolites associated with colorectal cancer using public databases

#### 3.3.1 Comparison of gut microbiota between CRC patients and healthy controls

At pretreatment, Shannon diversity was lower in patients than in healthy volunteers at both the genus level (Wilcoxon rank-sum *P* = 0.017; Figure 7A) and the species level (*P* = 0.0016; Figure 7B). Bray-Curtis community profiles differed between groups by ANOSIM at the genus level (*R* = 0.3118, *P* = 0.001; Figure 7C) and the species level (*R* = 0.2894, *P* = 0.001; Figure 7D). Because the groups were not matched for age or sex and no formal dispersion test is reported, these findings are presented as descriptive between-group differences rather than CRC-specific effects or evidence of greater within-group variability.

**Figure 7:**
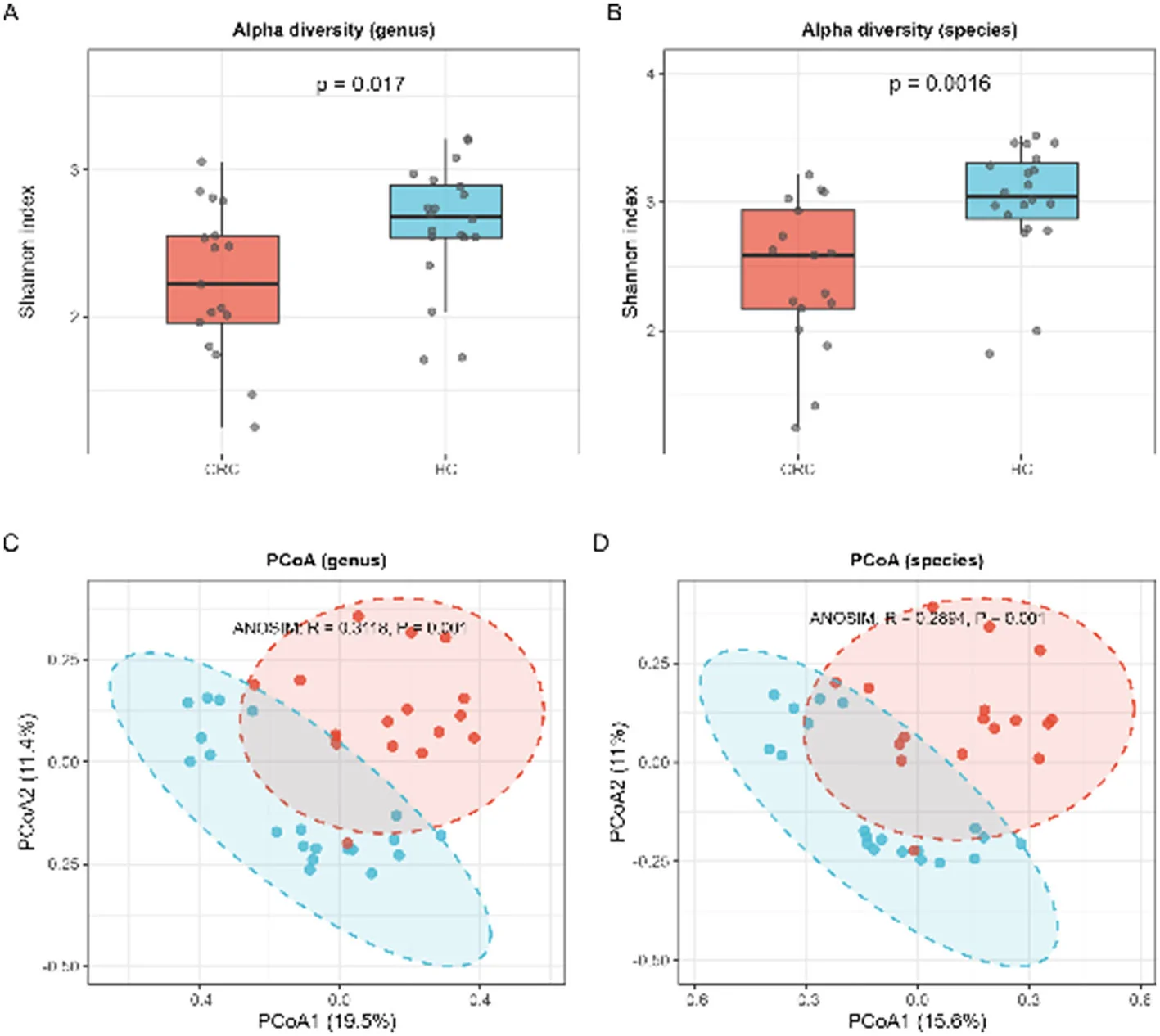
Descriptive comparison of gut microbiota between CRC patients and healthy controls. **(A)** Shannon index at the genus level (Wilcoxon rank-sum test, *P* = 0.017). **(B)** Shannon index at the species level (*P* = 0.0016). Boxes represent the interquartile range, with the horizontal line indicating the median. Each point represents one sample; red = CRC patients (*n* = 17), blue = healthy controls (*n* = 20). **(C)** Principal coordinate analysis (PCoA) based on Bray-Curtis distance at the genus level. **(D)** PCoA at the species level. Ellipses indicate 95% confidence intervals. Analysis of similarities (ANOSIM) with 999 permutations showed differences in microbial community structure between the two groups at both the genus level (*R* = 0.3118, *P* = 0.001) and the species level (*R* = 0.2894, *P* = 0.001). Because the groups were not matched for age or sex, these findings are descriptive and cannot be attributed to CRC alone.

Wilcoxon tests with Benjamini–Hochberg correction identified 21 genera and 12 species with different relative abundance between patients and healthy volunteers (FDR < 0.05; Supplementary Tables S2-1, S2-2). Patient samples had higher relative abundance of genera including *Bifidobacterium, Adlercreutzia, Peptostreptococcus, Gemella, Enterococcus*, and *Eggerthella*, and lower relative abundance of genera including *Achromobacter, Phocaeicola, Haemophilus*, and *Faecalibacillus*. At the species level, *Bifidobacterium longum, Adlercreutzia equolifaciens, P. stomatis*, and *G. morbillorum* were higher in patients, whereas *Haemophilus parainfluenzae* and *Phocaeicola plebeius* were lower. LEfSe yielded a broadly overlapping set of taxa (Figure 8); the complete results are shown in the figure rather than repeated in the text.

**Figure 8:**
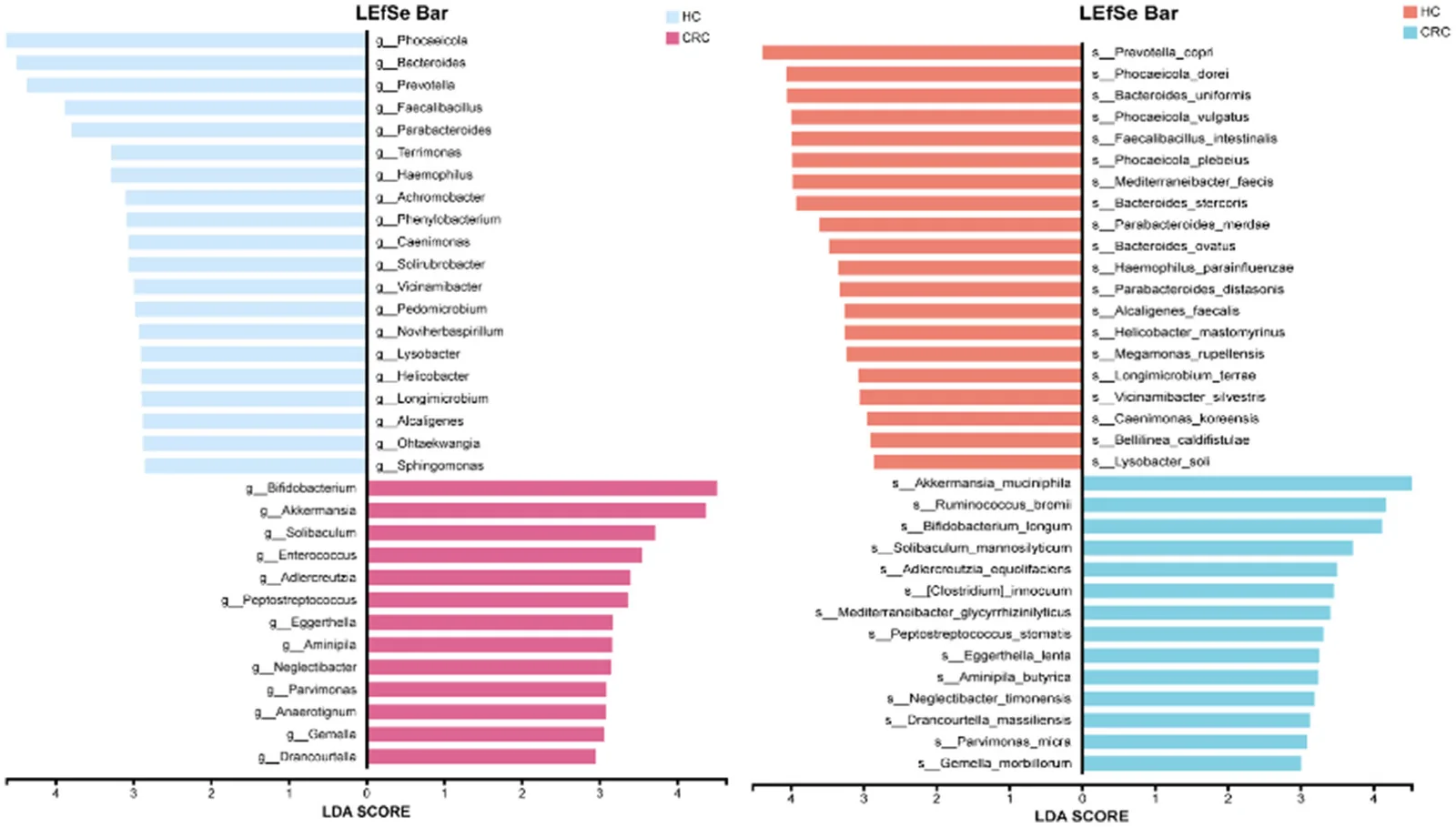
LEfSe analysis of differentially abundant taxa between CRC patients and healthy controls. Linear discriminant analysis (LDA) effect size (LEfSe) bar plots showing taxa with differential relative abundance between CRC patients and healthy controls. Left panel: genus-level taxa enriched in healthy controls (light blue) or CRC patients (pink). Right panel: species-level taxa enriched in healthy controls (red) or CRC patients (teal). The x-axis represents the LDA score (absolute value ≥ 2), and the y-axis lists taxa ordered by LDA score. *P* < 0.05 by the Kruskal–Wallis test was used as the significance threshold. LEfSe analysis was performed on the Majorbio Cloud Platform. Because the groups were not matched for age or sex, these findings are descriptive and cannot be attributed to CRC alone.

#### 3.3.2 Directional comparison with GMrepo

The patient-control taxa identified in this cohort were compared at the list level with CRC-associated taxa reported in GMrepo; raw sequence data were not pooled. Five species could be matched between the two sources, and all five had the same reported direction: *A. equolifaciens, P. stomatis*, and *G. morbillorum* were higher in CRC, whereas *H. parainfluenzae* and *P. plebeius* were lower (Supplementary Table S3). This result indicates directional consistency with heterogeneous published datasets and is not an independent validation of the present cohort.

In exploratory longitudinal models of these five species, *G. morbillorum* (estimate = −0.584, FDR = 1.35 × 10^−6^) and *P. stomatis* (estimate = −0.631, FDR = 4.42 × 10^−6^) decreased significantly during CCRT (Supplementary Table S4 and Figure 9). *A. equolifaciens, H. parainfluenzae*, and *P. plebeius* did not show significant time trends (all FDR > 0.05). Thus, case-control direction and longitudinal treatment-period change were concordant for two of the five matched species; the other three did not provide evidence of a treatment-period trend.

**Figure 9:**
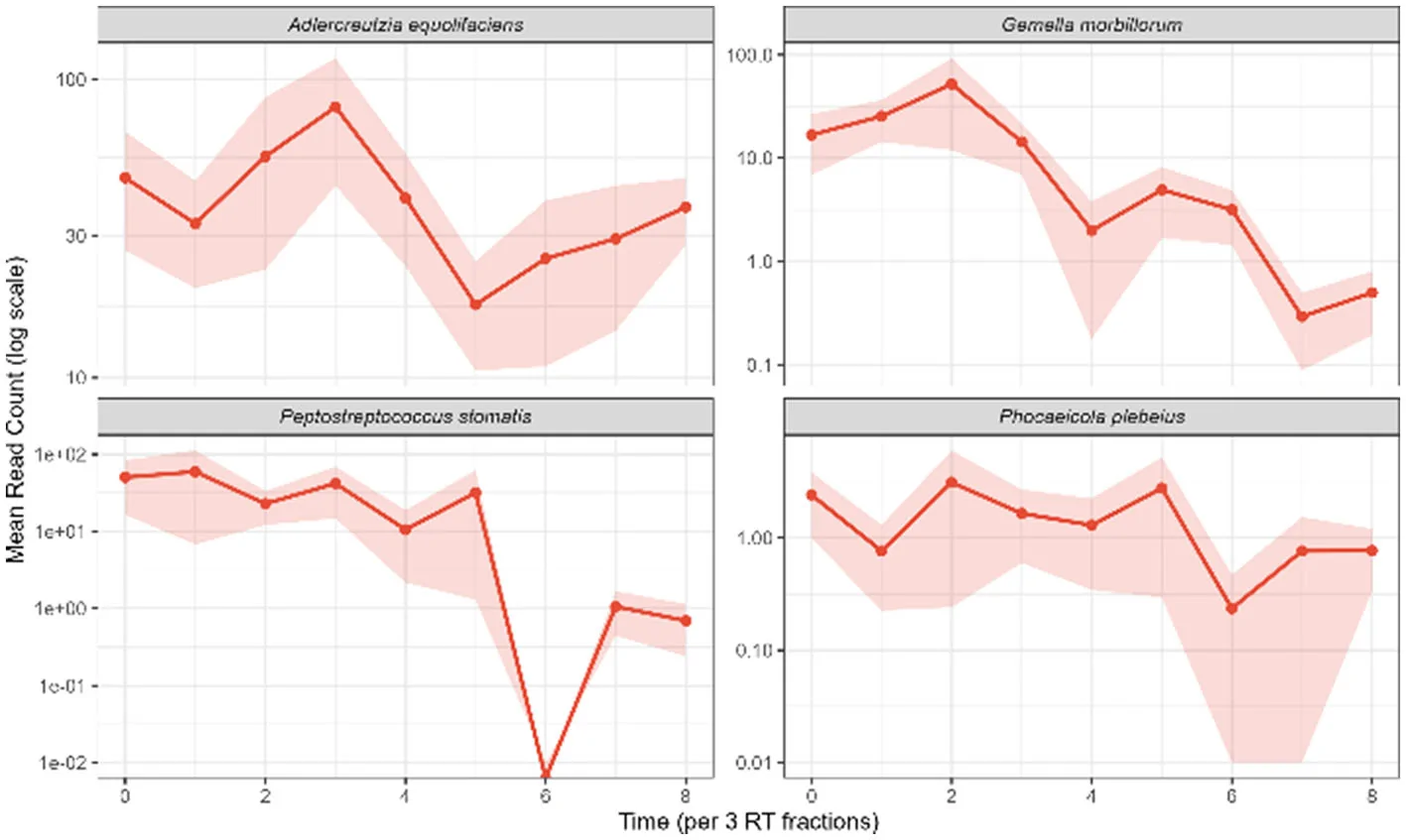
Temporal dynamics of four CRC-associated species during CCRT. Lines represent arithmetic means of raw read counts at each time point (every 3 radiotherapy fractions, 0–8), with shaded bands indicating standard error of the mean. The y-axis is on a log10 scale. The four species shown are among the 27 species with significant linear time trends after FDR correction. *Gemella morbillorum* (FDR = 1.35 × 10^−6^) and *Peptostreptococcus stomatis* (FDR = 4.42 × 10^−6^) had significant negative time coefficients, whereas *Adlercreutzia equolifaciens* and *Phocaeicola plebeius* did not show significant time trends (FDR > 0.05). These models describe changes in sequencing-depth-adjusted relative counts and do not establish absolute bacterial load changes.

## 4 Discussion

Alterations in the gut microbiota of patients with colorectal cancer have been reported across multiple cohorts (Jiang et al., 2026). However, most treatment studies have sampled the microbiome at only one or two time points, leaving the temporal course of microbial and metabolic changes during chemoradiotherapy incompletely characterized (Duan et al., 2025). We therefore used full-length 16S rRNA gene sequencing and untargeted metabolomics to profile fecal samples collected at nine planned time points from before treatment through capecitabine-based concurrent chemoradiotherapy. 19 patients were enrolled. Two were excluded from the longitudinal analysis because samples could not be obtained within the prespecified collection window spanning three radiotherapy fractions, leaving 17 patients in the analytic cohort. Importantly, the R0 sample was collected before both capecitabine and radiotherapy. Given the cohort size and observational design, the study was intended to identify candidate signals and temporal patterns rather than to test causal or predictive hypotheses.

Several microbial taxa and metabolites showed significant linear time trends during treatment. At the genus level, 19 genera changed over time, and 27 of 176 analyzed species showed significant trends after false discovery rate correction. Fifteen genera had significant, directionally concordant findings at the genus and species levels. *Parvimonas micra, Peptostreptococcus stomatis*, and *Gemella morbillorum* showed negative time trends, whereas *Lachnoclostridium pacaense, Blautia hydrogenotrophica, [Eubacterium] sulci*, and *Coprococcus eutactus* showed positive time trends. In parallel, 393 of 2,873 metabolite features changed significantly over time. These findings indicate multiple treatment-period changes at the level of individual taxa and metabolite signals, but they do not by themselves establish a global restoration of the microbial community or a beneficial functional shift.

The decreases in *P. micra, P. stomatis*, and *G. morbillorum* are of particular interest because these species have previously been reported to be enriched in colorectal cancer cohorts (Chang et al., 2023). The case-control analysis in the present study showed the same direction for *P. stomatis* and *G. morbillorum*, and both species also matched the direction of colorectal cancer associations curated in GMrepo (Camañes-Gonzalvo et al., 2024). Their subsequent negative time trends therefore provide internally coherent evidence that some taxa associated with colorectal cancer changed during chemoradiotherapy. This observation should nevertheless be interpreted narrowly. A match to a curated database is a directional comparison, not independent validation, and relative sequencing counts cannot demonstrate eradication or an absolute reduction in bacterial load. Moreover, only two of the five species that could be matched to GMrepo showed significant longitudinal trends; *Adlercreutzia equolifaciens, Haemophilus parainfluenzae*, and *Phocaeicola plebeius* did not. Disease association and treatment responsiveness are therefore not interchangeable properties.

The species with positive time trends also require cautious interpretation. *L. pacaense, B. hydrogenotrophica, [Eubacterium] sulci*, and *C. eutactus* have been linked in previous studies to fermentation or short-chain fatty acid-related metabolic capacity. In the present study, however, neither short-chain fatty acids nor microbial functional genes or transcriptional activity was measured. An increase in relative abundance therefore cannot be taken as evidence that short-chain fatty acid production increased or that mucosal function improved (Koutoukidis et al., 2022). These taxa are better described as species with previously reported metabolic potential that increased relative to the remainder of the measured community. Shotgun metagenomics, absolute microbial quantification, and targeted measurement of acetate, propionate, and butyrate will be required to determine whether the observed taxonomic changes correspond to functional changes.

The metabolomics results further demonstrate that the treatment period was accompanied by changes in the fecal metabolic environment. Among the most prominent positive trends were lumirubin and chenodeoxycholic acid, whereas several annotated phospholipids, including PS (18:2/14:0), PC (LTE4/15:0), and DG (18:4/14:1), showed negative trends. These observations support focused hypotheses involving bilirubin products, bile acids, and membrane-lipid-related metabolites. They do not establish coordinated activation or suppression of entire metabolic pathways. Of the 393 significant metabolite features, only 103 had KEGG identifiers. Primary bile acid biosynthesis, glycerophospholipid metabolism, and glycine, serine, and threonine metabolism met the nominal *P*-value threshold, but none remained significant after false discovery rate correction. The pathway results should therefore be reported as exploratory prioritization signals rather than confirmed pathway-level findings.

The integrated analysis identified 17 of 50 selected species-metabolite pairs that remained significant in mixed-effects models containing time and a patient-specific random intercept. The positive association between *C. eutactus* and stercobilin, the negative association between Escherichia and stercobilin, and the negative association between *Ruminococcus gnavus* and prostaglandin F3a illustrate that different taxa can show distinct relationships with metabolites that change during treatment. These associations generate experimentally testable candidates, but they do not define a microbe-pathway-metabolite axis. The candidate pairs were first selected according to correlation strength and then tested in the same dataset, which can produce optimistic estimates after selection. Residual correlation may also persist when both variables share nonlinear or patient-specific temporal trajectories. Independent replication prespecified pair testing, and analyses that separate within-patient from between-patient covariation are needed before directionality or mediation is considered.

The pretreatment comparison provided a separate view of microbial differences between patients with rectal cancer and healthy volunteers. Patients had lower Shannon diversity at both the genus and species levels, and Bray-Curtis profiles differed between groups by ANOSIM. Several taxa previously associated with colorectal cancer, including *P. micra, P. stomatis*, and *G. morbillorum*, were enriched in patients. Other findings were more heterogeneous. *Bifidobacterium longum* and *Akkermansia muciniphila* were also enriched, whereas *Phocaeicola vulgatus, Haemophilus parainfluenzae, Phocaeicola plebeius*, and *Prevotella copri* were depleted. This mixture argues against classifying the observed community simply as a loss of beneficial organisms and an expansion of pathogens. *A. muciniphila* and *P. copri*, in particular, have shown context-dependent associations across populations and disease settings (Ahmed, 2024). Differences in diet, geography, intestinal transit, medication exposure, and disease characteristics may contribute, but these explanations were not tested in this cohort (Ruple et al., 2026).

The case-control results should also be separated conceptually from the longitudinal treatment analysis. The healthy volunteers were substantially younger than the patients and were not individually matched. Diet, body mass index, bowel habits, medication use, and other potential confounders were not comprehensively incorporated into the comparison. Consequently, the between-group findings cannot be attributed solely to rectal cancer. In addition, ANOSIM can be influenced by differences in dispersion within-groups. The apparently greater dissimilarity among patients should not be interpreted as increased inter-individual variability unless it is supported by a formal dispersion test such as PERMDISP. These limitations do not negate the observed differences, but they restrict their value to descriptive comparison and candidate selection.

The dense sampling design is the main strength of this study. Collection from a true pretreatment R0 point and at repeated intervals during therapy allowed early and sustained trends to be examined within the same patients. Full-length 16S rRNA gene sequencing improved taxonomic resolution relative to short-amplicon approaches, and integration with metabolomics enabled candidate relationships to be evaluated while accounting for repeated measurements. The inclusion of false discovery rate correction for the principal taxonomic and metabolite analyses also reduced the risk of interpreting isolated nominal *P* values. The exclusion of two of 19 enrolled patients because the required collection windows could not be met nevertheless shows that this schedule places a practical burden on participants. Future studies should report the number of available samples at every time point and use prespecified strategies for missing data so that feasibility and potential selection bias can be assessed.

Several additional limitations affect interpretation. First, this was a single-center study with 17 patients in the final longitudinal analysis. The estimates are therefore imprecise and may not generalize to other populations, treatment schedules, or supportive-care practices. Second, because capecitabine and radiotherapy were administered as a combined regimen, their separate effects cannot be distinguished, even though R0 preceded both treatments. Treatment-associated changes may also reflect concurrent changes in diet, stool transit, symptoms, medications, or supportive care. Third, time was modeled as a linear numerical variable. Early peaks, late plateaus, or recovery patterns could be missed, and future analyses should compare linear models with spline, categorical-time, or other prespecified nonlinear models. Fourth, 16S rRNA sequencing produces compositional relative-abundance data and cannot establish absolute bacterial expansion or depletion. Species-level assignment and functional inference remain imperfect even with full-length sequencing.

Fifth, the untargeted metabolomics platform is subject to feature-annotation uncertainty, and pathway analysis was limited by incomplete KEGG mapping and the small number of hits in each pathway. Key compounds, including bile acids, bilirubin products, and representative phospholipids, require confirmation using authentic standards and targeted quantitative assays. Sixth, the microbe-metabolite analysis involved data-dependent candidate selection and lacked an independent validation cohort. Seventh, the study did not relate microbial or metabolic trajectories to prespecified clinical endpoints such as tumor regression grade, pathological complete response, radiographic response, diarrhea, radiation enteritis, or hematologic toxicity. The results therefore cannot be interpreted as predictive biomarkers or used to support microbiome-directed treatment. Finally, the unmatched healthy control group and incompletely measured confounders limit disease-specific interpretation of the cross-sectional findings.

These results define several priorities for subsequent work. A multicenter prospective study should retain a pretreatment sample before any therapy, use feasible and prespecified sampling windows, document missingness, and collect detailed information on diet, medications, bowel function, and supportive care. Clinical response and toxicity endpoints should be defined before analysis, with sample size based on the expected longitudinal effect and attrition. Absolute microbial quantification and shotgun metagenomics should be combined with targeted measurement of short-chain fatty acids, bile acids, stercobilin, and selected phospholipids. Candidate species-metabolite pairs should be prespecified in a discovery cohort and tested in an independent cohort. Only associations that replicate and precede clinical outcomes should progress to mechanistic experiments using bacterial isolates, metabolite supplementation or inhibition, organoid models, or appropriately designed animal studies.

In summary, this longitudinal multi-omics study identified taxonomic and fecal metabolite signals that changed from a pretreatment baseline during capecitabine-based chemoradiotherapy for rectal cancer. The most consistent observations were negative time trends in several species previously associated with colorectal cancer, positive trends in several species with reported fermentative potential, and changes in selected metabolites related to bile acids, bilirubin, and phospholipids. The pathway-level and species-metabolite findings remain exploratory, and the study did not test whether these changes influence treatment response or toxicity. Independent, adequately powered studies with absolute quantification, targeted metabolomics, clinical endpoints, and external validation are required before these candidates can be considered for biomarker development or microbiome-directed intervention.

## Data Availability

The raw 16S rRNA gene sequencing data are available in the European Nucleotide Archive (ENA) under BioProject accession number PRJEB127908 (https://www.ebi.ac.uk/ena/browser/view/PRJEB127908). The untargeted metabolomics data are available in MetaboLights under accession number MTBLS15915 (https://www.ebi.ac.uk/metabolights/MTBLS15915).
